# Supplementary material for: Identification of photoperiod-induced specific miRNAs in the adrenal glands of Sunite sheep (Ovis aries)
Source: Front Vet Sci. 2022 Jul 22;9:888207. doi: 10.3389/fvets.2022.888207 (PMC9354845; doi:10.3389/fvets.2022.888207)
Supplement: Supplementary file 1 [file Data_Sheet_1.ZIP › Supplementary file/miRDeep2 file.zip!/miRDeep2 file/Novel_Structure/readme.doc]

1、各文件夹下PDF文件，是各个样本中识别为未知miRNA的图示结果，其中展示了识别到novel miRNA前体二级结构，对应的count数和reads序列。
